# Newborn screen metabolic panels reflect the impact of common disorders of pregnancy

**DOI:** 10.1038/s41390-021-01753-7

**Published:** 2021-10-20

**Authors:** Jonathan D. Reiss, Alan L. Chang, Jonathan A. Mayo, Katherine Bianco, Henry C. Lee, David K. Stevenson, Gary M. Shaw, Nima Aghaeepour, Karl G. Sylvester

**Affiliations:** 1 Division of Neonatology, Department of Pediatrics, Stanford University School of Medicine, Palo Alto, CA, USA.; 2 Department of Anesthesiology, Pain and Perioperative Medicine, Stanford University, Palo Alto, CA, USA.; 3 March of Dimes Prematurity Research Center, Stanford University School of Medicine, Palo Alto, CA, USA.; 4 Division of Maternal-Fetal Medicine, Department of Obstetrics & Gynecology, Stanford University School of Medicine, Palo Alto, CA, USA.; 5 California Perinatal Quality Care Collaborative, Palo Alto, CA, USA.; 6 Division of Pediatric Surgery, Department of Surgery, Stanford University School of Medicine, Palo Alto, CA, USA.; 7 These authors contributed equally: Jonathan D. Reiss, Alan L. Chang.; 8 These authors jointly supervised this work: Gary M. Shaw, Nima Aghaeepour, Karl G. Sylvester.

## Abstract

**BACKGROUND:**

Hypertensive disorders of pregnancy and maternal diabetes profoundly affect fetal and newborn growth, yet disturbances in intermediate metabolism and relevant mediators of fetal growth alterations remain poorly defined. We sought to determine whether there are distinct newborn screen metabolic patterns among newborns affected by maternal hypertensive disorders or diabetes in utero.

**METHODS:**

A retrospective observational study investigating distinct newborn screen metabolites in conjunction with data linked to birth and hospitalization records in the state of California between 2005 and 2010.

**RESULTS:**

A total of 41,333 maternal–infant dyads were included. Infants of diabetic mothers demonstrated associations with short-chain acylcarnitines and free carnitine. Infants born to mothers with preeclampsia with severe features and chronic hypertension with superimposed preeclampsia had alterations in acetylcarnitine, free carnitine, and ornithine levels. These results were further accentuated by size for gestational age designations.

**CONCLUSIONS:**

Infants of diabetic mothers demonstrate metabolic signs of incomplete beta oxidation and altered lipid metabolism. Infants of mothers with hypertensive disorders of pregnancy carry analyte signals that may reflect oxidative stress via altered nitric oxide signaling. The newborn screen analyte composition is influenced by the presence of these maternal conditions and is further associated with the newborn size designation at birth.

## BACKGROUND

The newborn screen (NBS), obtained via heel stick dried blood spot collection, is a population metabolic screening test for inborn errors of metabolism conducted by individual states on all newborns shortly after birth.^1^ As such, the NBS reflects the molecular phenotypic change among analytes of intermediate metabolism caused by underlying biologic derangements that are heritable. The NBS has also been used as a means of identifying altered metabolic states of the newborn associated with clinical disease unrelated to primary genetic or metabolic diagnoses.^2–10^ These studies demonstrate that the NBS can yield important insights on metabolic disturbances associated with conditions other than genetic inborn errors of metabolism, including gestational dating, and the risks of acquired diseases of the newborn, including sepsis, respiratory distress syndrome, hyper-bilirubinemia, persistent pulmonary hypertension, necrotizing enterocolitis (NEC), hypoxic–ischemic encephalopathy, and mortality. However, the impact of common maternal conditions such as diabetes and preeclampsia that may affect newborn metabolic function as reflected in the NBS has not been widely explored.^11^ This knowledge gap is important as the NBS metabolic composition is influenced by the intrauterine environment and immediate postnatal transition to extrauterine life.^11–13^ Although there are studies that have evaluated the metabolic composition of newborns vis-à-vis the newborn growth status,^14,15^ we are not aware of prior investigations that assess the metabolite composition of the newborn via the NBS for normal and growth-altered states secondary to maternal conditions.

The effects of maternal diabetes and hypertensive disorders on the fetus are often reflected in growth parameters that are measured throughout gestation and at birth.^16^ Growth as measured by anthropometric indices is largely defined by epidemiologic standards and reference curves^17–20^ that help clinicians weigh clinical risk of disease.^21^ For the fetus and newborn, the maternal diabetic and hypertensive disorder phenotypes are most profoundly expressed via metabolic-dependent processes that manifest in extremes of newborn size and include large for gestational age (LGA) and small for gestational age (SGA) designations.^22,23^ The birth weight for age designation of the newborn is critical, as significant short- and long-term morbidity and mortality risk occurs secondary to in utero growth disturbances. In addition, extremes of birth weight place the infant at heightened risk for adult metabolic disease.^24^ Despite these sequelae, little is understood about the relationship between maternal disorders of pregnancy, infant size, and effects on the newborn metabolome.

The objective of this study was to determine whether there are distinct NBS metabolic patterns among newborns affected by maternal hypertensive disorders and diabetes in utero. We hypothesized that the effects of maternal diabetes and hypertension on newborn metabolism and clinical growth phenotypes, manifested as birth weight, can be quantified using universally applied NBS analytes. Additionally, we posited that the NBS analyte composition will enable us to develop a metabolic model of fetal growth outliers. These findings are potentially significant as greater mechanistic understanding of the means by which the intrauterine environment contributes to newborn biologic vulnerability for acquired diseases associated with the growth altered fetus is needed.

## METHODS

We analyzed 41,333 singleton live births from a total of 3,175,992 singleton live births in California between 2005 and 2010 by merging California Perinatal Quality Care Collaborative (CPQCC) and California Office of Statewide Health Planning and Development (OSHPD) birth and hospitalization records with California Biobank NBS analyte data (Supplemental Fig. 1). NBS collection in California is mandatory for all newborns, with 80 different genetic and congenital disorders screened for. The NBS analytes measured via tandem mass spectrometry includes acylcarnitines, amino acids, and ratios of analytes as visible in Supplemental Table 1. For patients born between January 2005 and November 2009, all newborns had at least one blood spot collection for newborn screening performed between 12 h and 8 days after birth. For infants born between December 2009 and December 2010, data on timing of collection were not available. Data regarding blood transfusions, missed NBSs, and other variables related to timing of collection were also not available. The California Biobank Program is the biospecimen and data repository of the California Genetic Disease Screening Program (GDSP). This program oversees and administers the NBS. Use of these data was approved by the California Committee for the Protection of Human Subjects, CPQCC, and the Stanford University Institutional Review Board.

Records obtained from the California GDSP included live born infants at 20–29 weeks gestation, a proportion of whom carried diagnoses of NEC, retinopathy of prematurity (ROP), bronchopulmonary dysplasia (BPD), and intraventricular hemorrhage (IVH). Records also included a random sampling of liveborn infants born at 30–44 weeks gestation, none of whom carried diagnoses of NEC, ROP, BPD, and IVH. From this case–control collection of infants, we obtained NBS metabolite measurement values from the California State Biobank and linked this with demographic information and clinical data for both the newborn and the mother obtained from OSHPD and CPQCC. These pooled records favored inclusion of premature infants, as 33% of the study population included infants born at <37 weeks gestation. This enabled us to more robustly examine for the presence or absence of maternal conditions that may lead to early preterm birth such as preeclampsia and more easily compare preterm and term populations. Certain demographic and clinical data elements were converted to categorical form. Additionally, gestational age records were converted to 2-week intervals and birth weights were converted to 50-g intervals. All forms of diabetes and hypertensive disorders were defined using International Classification of Diseases (ICD)-9 code definitions that were part of the hospital record (Supplemental Table 2). Size for age definitions at birth were based on World Health Organization records of normative data for fetal size.^25^

To determine NBS analyte associations with maternal outcomes of interest including maternal diabetes and hypertensive disorders of pregnancy, we used elastic net logistic regression modeling with repeated tenfold cross-validation. The elastic net modeling assumed statistical independence between all metabolite variables enabling us to observe metabolites reflective of a given maternal condition. NBS metabolite values were used as input features for elastic net logistic regression. Binary indicators were used for the presence or absence of a given maternal outcome and were subsequently used as labels for model training. Controls were defined as mothers who did not have ICD-9 code definitions of any form of diabetes or hypertensive disorder of pregnancy. Hyperparameter tuning was performed on an inner *K*-fold cross-validation loop on the training set to select an optimal strength of regularization for metabolite feature selection using area under the receiver-operator curve (AUROC) as the selection criteria for hyperparameter values. The optimal hyperparameters were then used in a new model that was evaluated on the test set. Performance was evaluated on the basis of AUROC for model predictions made on test set samples. Model coefficients were collected at the end of each tenfold cross-validation iteration to identify metabolites that were the most predictive in identifying infants from mothers with various conditions. For analysis of SGA and LGA outcomes of infants from mothers with hypertensive or diabetic disorders of pregnancy, additional regression correction was performed for the potentially confounding covariates of cigarette smoking, body mass index, gestational age, and maternal age. Models were then trained using residual metabolite values after covariate correction.

## RESULTS

**Fig. 1:**
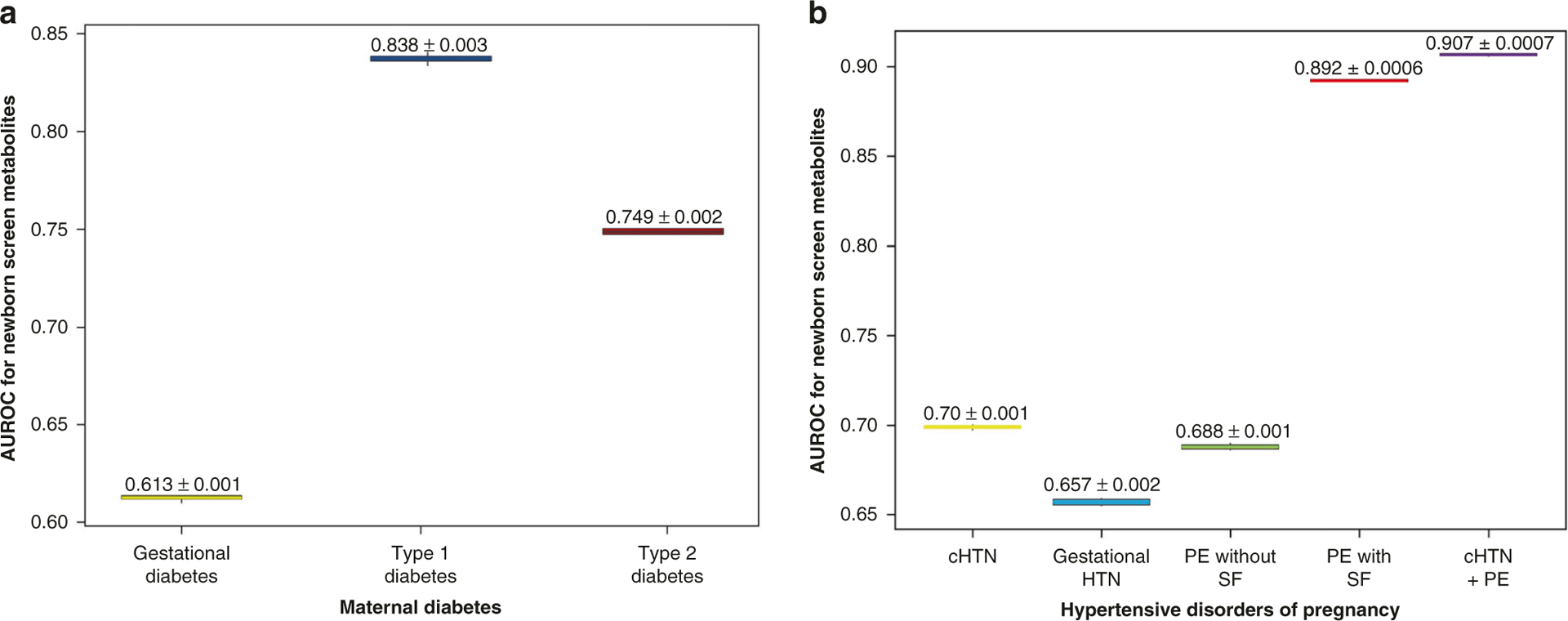
Elastic net model performance for maternal conditions including diabetes and hypertensive disorders of pregnancy. Box-and-whisker plots reflect elastic net performance modeling of NBS analytes to reflect (**a**) maternal diabetes and (**b**) hypertensive disorders of pregnancy via AUROC values. Metabolites were most predictive of Type 1 Diabetes and cHTN + PE conditions. Plots reflect interquartile range with whiskers extending from hinges to the greatest value no larger than 1.5 * the interquartile range. Box-and-whisker ranges reflect repeated modeling 10 times. AUROC area under the receiver-operating characteristic, HTN hypertension, cHTN chronic hypertension, PE preeclampsia, SF severe features, cHTN + PE chronic hypertension with superimposed preeclampsia with or without severe features.

The maternal and neonatal characteristics from the dataset are depicted in Supplemental Tables 3 and 4. All mothers with any form of diabetes were mutually exclusive of one another such that, if a mother was diagnosed with gestational diabetes, she did not have concurrent type 1 or type 2 diabetes. This was also the case for mothers diagnosed with either hypertension or preeclampsia. The relationship between infants’ NBS analytes and maternal diabetes or hypertensive disorders was estimated by the mean area under the AUROC curve as demonstrated in Fig. 1. As elastic net excels in prediction, Fig. 1 describes the relative predictive power of the NBS metabolites alone as measured by AUROC values for each of the maternal outcomes of interest. An AUROC value of 0.5 is suggestive of no discriminatory capability or predictive power. Conversely, values that approach zero or one suggest maximum negative or positive predictive power. Among mothers with diabetes, the NBS metabolites of infants exposed to type 1 diabetes best reflected this condition with a mean AUROC = 0.838 ± 0.003. For mothers with a hypertensive disorder of pregnancy, the NBS metabolites of infants exposed to chronic hypertension with superimposed preeclampsia best reflected this condition with a mean AUROC = 0.907 ± 0.0007. All maternal conditions had corresponding NBS metabolite values that uniquely reflected a given exposure as demonstrated by each mean AUROC value observed in Fig. 1.

**Fig. 2:**
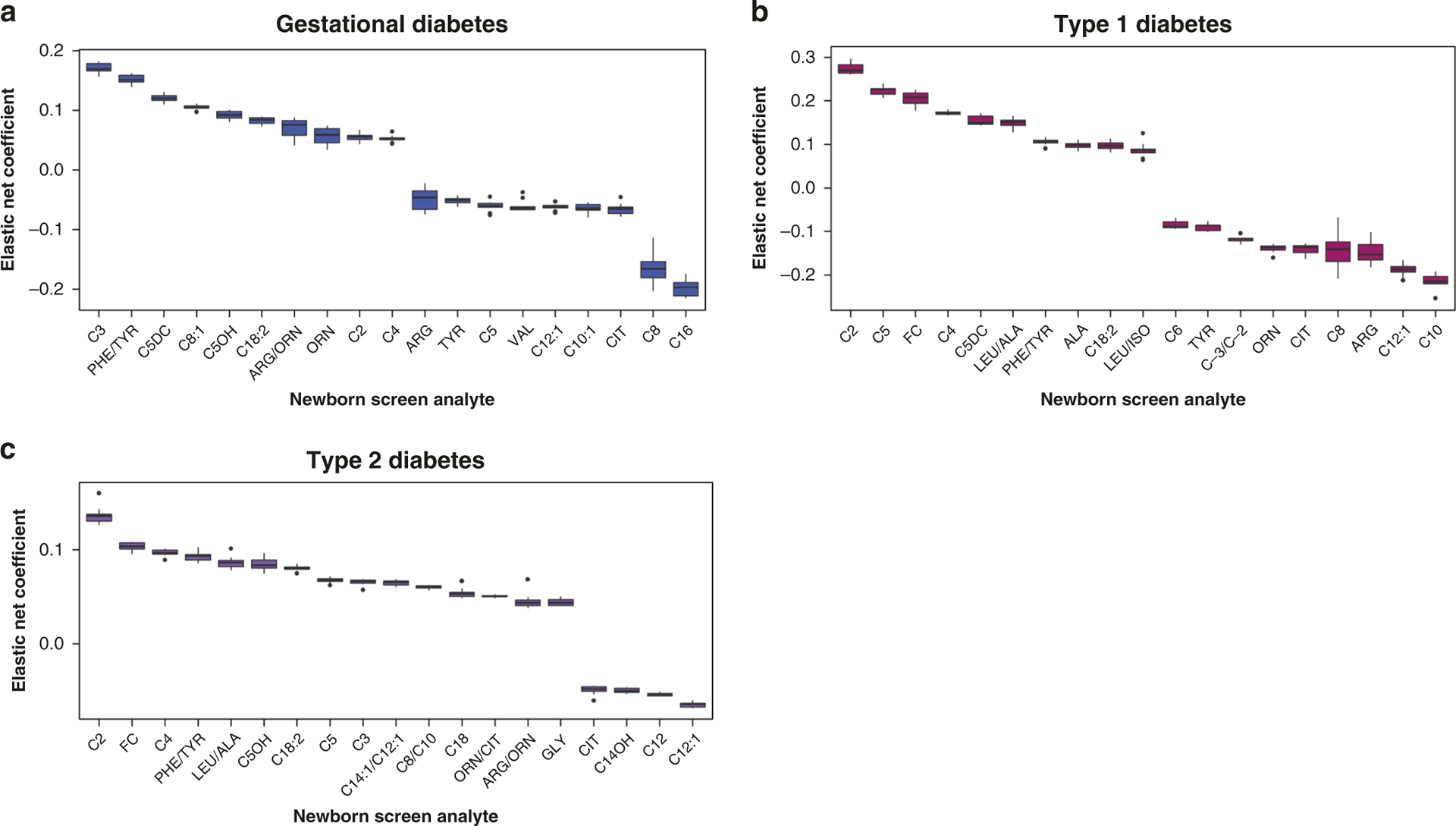
Box-and-whisker elastic net model coefficients for gestational diabetes, type 1 diabetes, and type 2 diabetes. Coefficients are shown that demonstrate the relative importance and predictive power for each metabolite utilizing elastic net regression models trained to identify cases of (**a**) gestational diabetes, (**b**) type 1 diabetes and (**c**) type 2 diabetes from newborn screening metabolite measurements. Box-and-whisker plots reflect interquartile range with whiskers extending from hinges to the greatest value no larger than 1.5 * the interquartile range. Box-and-whisker ranges reflect repeated modeling for 10 iterations. Only metabolites with coefficients highly predictive of any form of diabetes were included. Abbreviations for newborn screen metabolites can be found in Supplemental Table 1.

Individual NBS metabolic analytes were also used to develop a model via elastic net regression to identify mothers with analytes reflective of all forms of diabetes, including gestational diabetes, type 1 diabetes, or type 2 diabetes. Figure 2 describes the importance (coefficients) and predictive power of the metabolites in relation to one another for each individual model. For instance, a coefficient of 0.2 is twice as important for model prediction as a coefficient of 0.1. Only metabolites with the largest absolute coefficient values were ultimately selected. Analytes are grouped according to model coefficient values. All coefficients greater than or less than zero correspond with positive or negative predictive capabilities. A coefficient value close to zero suggests minimal predictive capability. Short-chain acylcarnitines C2, C3, C4, and C5 were predictive of gestational, type 1, and type 2 diabetes in varying degrees. Free carnitine was also predictive of type 1 and type 2 diabetes.

**Fig. 3:**
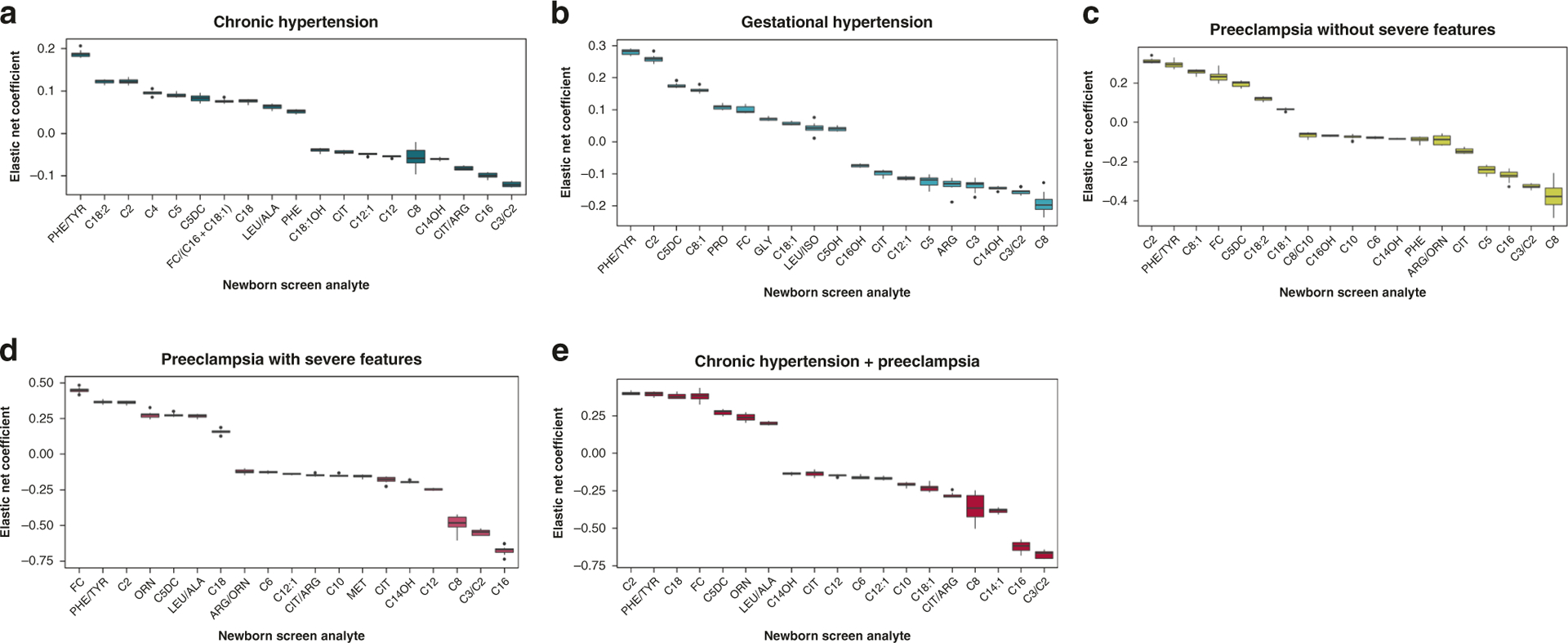
Box-and-whisker elastic net model coefficients for hypertensive disorders of pregnancy. **a** Chronic hypertension, **b** gestational hypertension, **c** preeclampsia without severe features, **d** preeclampsia with severe features, **e** chronic hypertension with superimposed preeclampsia (with or without severe features). Coefficients are shown that demonstrate the relative importance and predictive power for each metabolite utilizing elastic net regression models trained to identify cases of hypertensive disorders of pregnancy from newborn screening metabolite measurements (**a**–**e**). Box-and-whisker plots reflect interquartile range with whiskers extending from hinges to the greatest value no larger than 1.5 * the interquartile range. Box-and-whisker ranges reflect repeated modeling for 10 iterations. Only metabolites with coefficients highly predictive of any form of diabetes were included. Abbreviations for newborn screen metabolites can be found in Supplemental Table 1.

Similarly, NBS analytes were used to develop a model of maternal hypertensive disorders (Fig. 3). For the preeclampsia without severe features, preeclampsia with severe features, and chronic hypertension with superimposed preeclampsia cohorts, C2, FC, C5-DC, C18, and ornithine were notable analytes positively predictive for the presence of disease. Citrulline was negatively predictive in cases of preeclampsia without severe features and preeclampsia with severe features. Additional analytes were positively and negatively predictive with various forms of hypertension or preeclampsia as visible in Fig. 3.

**Fig. 4:**
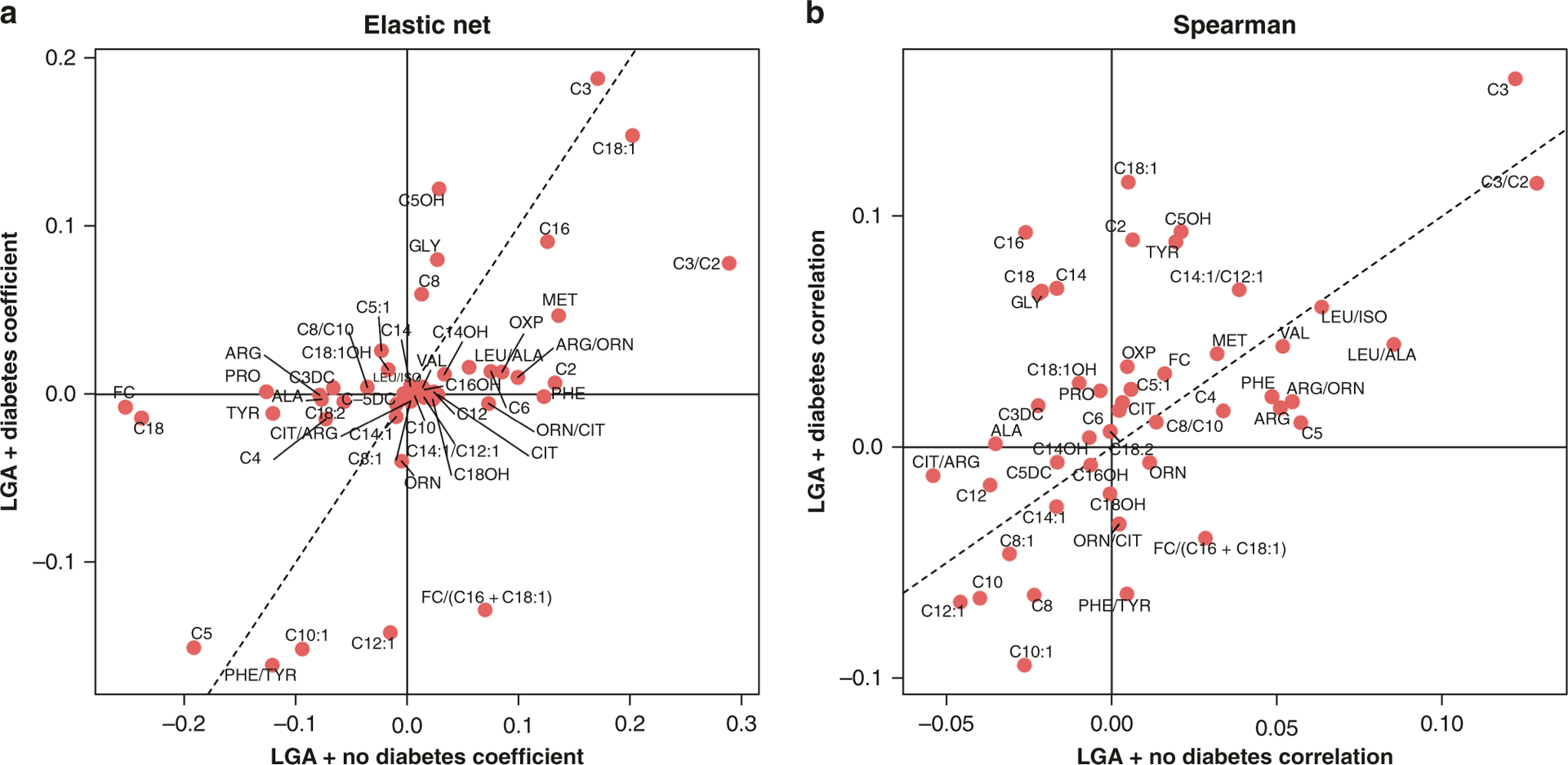
Comparison of LGA infants with and without diabetes (gestational diabetes, Type 1, or Type 2) between elastic net and Spearman correlation. A comparison of LGA infants with (*y*-axis) and without (*x*-axis) exposure to maternal diabetes. The dotted line signifies coefficient or correlation values that are identical for infants born LGA either with or without exposure to maternal diabetes and suggests no discriminatory capability between the neonates exposed to diabetes and those without exposure. Metabolite positions near the center of each figure reflect less predictive capability. Metabolite positions distant from the *x*- or *y*-axis suggest greater predictive power (elastic net) or association (Spearman). **a** Elastic Net metabolites predictive of LGA + exposure to maternal diabetes include C5OH, GLY and C8. Metabolites suggestive of negative predictive ability for LGA + exposure to maternal diabetes include C12:1 and C10:1. Additionally, metabolites with negative predictive power for LGA without exposure to maternal diabetes include FC and C18. Metabolites with positive predictive power for this cohort include PHE and C2. **b** Utilizing Spearman correlation, metabolites positively associated with LGA + exposure to maternal diabetes include C18:1, C2, TYR, C5OH, C14, C16, C18, and GLY. Metabolites negatively associated with LGA + Diabetes include C8, C10, C10:1, C12:1. Metabolites positively associated for LGA without exposure to diabetes include C5, ARG and PHE. Metabolites negatively associated for LGA without exposure to diabetes include ALA and C12. LGA large for gestational age.

**Fig. 5:**
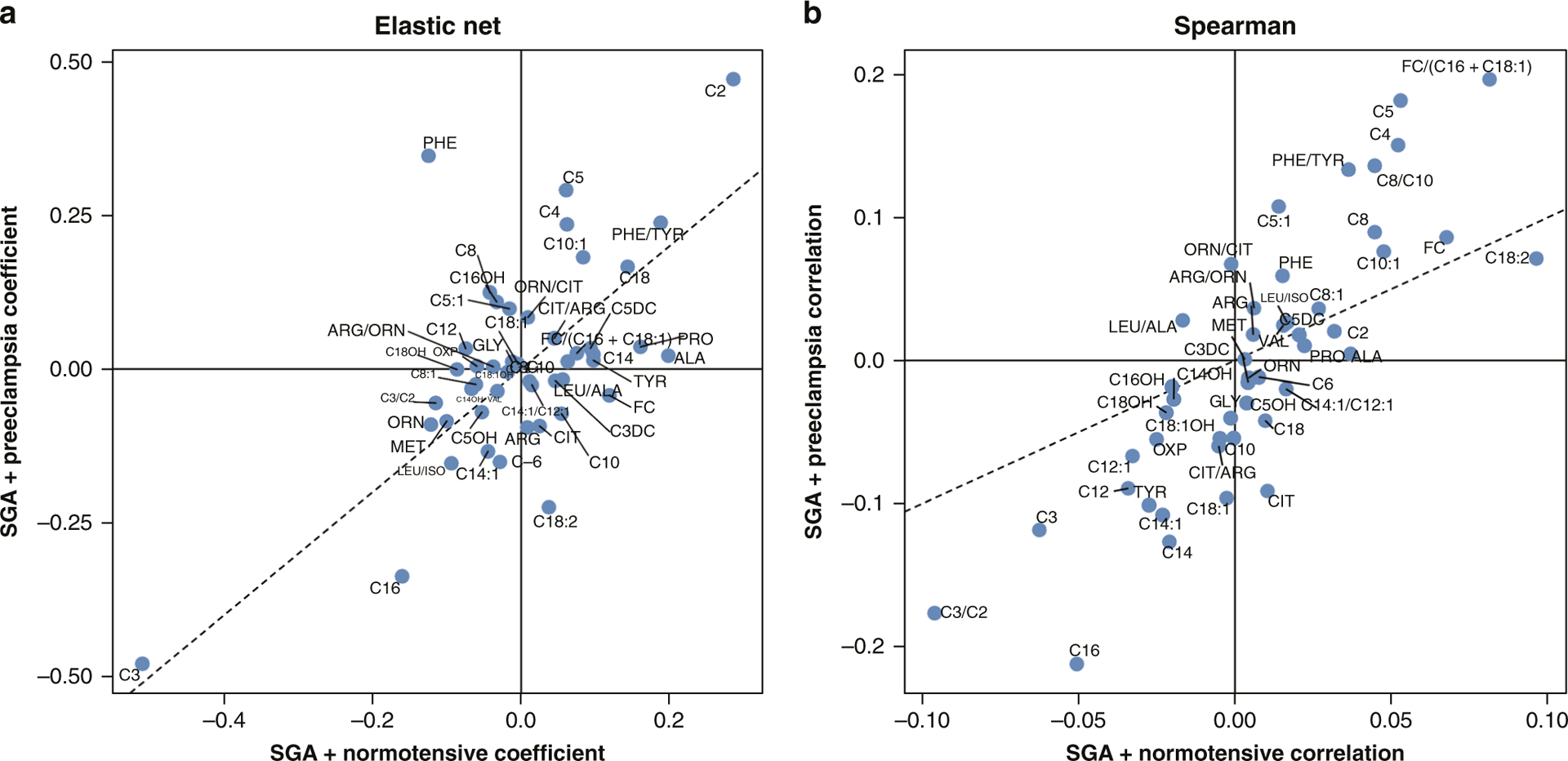
SGA infants with and without preeclampsia comparing elastic net and Spearman correlation. A comparison of SGA infants with (*y*-axis) and without (*x*-axis) exposure to preeclampsia. The dotted line signifies coefficient or correlation values that are identical for infants born SGA either with or without exposure to maternal preeclampsia and suggests no discriminatory capability between the neonates exposed to preeclampsia and those without exposure. Metabolite positions near the center of each figure are suggestive of minimal predictive ability or association. Metabolite positions distant from the *x*- or *y*-axis suggest greater predictive power (Elastic Net) or association (Spearman). **a** Elastic Net metabolites predictive of SGA + exposure to maternal preeclampsia include PHE, C2, C4, C5 and C10:1. Metabolites with negative predictive power include C16 and C18:2. **b** Utilizing Spearman correlation metabolites positively associated with SGA + exposure to maternal preeclampsia include C4, C5, C5:1 and C8. Metabolites negatively associated with SGA + exposure to maternal preeclampsia include C14, C14:1, C16, C18:1, and CIT. Metabolites positively associated with SGA without preeclampsia exposure include ALA and PRO. SGA Small for Gestational Age, Preeclampsia = combination of all forms of preeclampsia including chronic hypertension with superimposed preeclampsia, preeclampsia without severe features, and preeclampsia with severe features.

Further, the variable of infant size for gestational age was introduced to the model and overlaid with our prior modeling for diabetes and hypertensive disorders to determine whether there were analytes specific to the LGA designation with and without diabetes. Figure 4 includes plots for LGA infants delivered to mothers without diabetes (*x*-axis) and LGA infants with any form of diabetes (*y*-axis). Elastic net regression was used to identify predictive analytes implicated, with Spearman correlation coefficients used for validation. Notable analytes associated and predictive of LGA infants born to diabetic mothers include glycine, C5-OH, C3, and long-chain acylcarnitines C14, C16, C18, and C18:1. Figure 5 includes plots for SGA infants with and without any form of preeclampsia, including cases of preeclampsia without severe features, with severe features, and superimposed on chronic hypertension. For SGA infants born to mothers with any form of preeclampsia, notable analytes implicated in both the elastic net regression prediction model and the Spearman correlation values include phenylalanine, citrulline, C5:1, C4, and C5. Arginine was positively associated and ornithine and citrulline were negatively associated with being born SGA to a mother with any form of preeclampsia in the Spearman correlation.

## DISCUSSION

To our knowledge, this study is the largest to demonstrate that there are characteristic metabolic signatures observed in newborns who have been delivered by mothers who had common conditions of pregnancy, including hypertensive and diabetic disorders. This study provides at least three novel findings. First, the metabolic signs of insulin resistance are already present in infants born to diabetic mothers, as evidenced by positive associations with short-chain acylcarnitines and free carnitine, both of which have been found to be elevated in various metabolomic studies in animal models and patients with diabetes.^26–29^ Second, for infants born to mothers with a hypertensive disorder of pregnancy, there are significant changes in analytes that may promote oxidative stress via altered nitric oxide signaling and abnormal lipid metabolism. Metabolites implicated in these pathways include C2, ornithine, citrulline, and free carnitine.^30–33^ Lastly, the analyte composition in the newborn is associated with a growth phenotype (i.e., SGA or LGA), and this association is further influenced by the presence or absence of maternal diabetes or hypertension. We posit that with further investigation the analyte composition may act as a biological taxonomy for the growth altered fetus, extending the definitions of the SGA and LGA newborn and associated clinical risk by reflecting underlying biological processes, rather than relying on anthropometric indices alone.

Few prior studies have examined NBS metabolites to identify metabolic signatures associated with pregnancy disorders that also affect the newborn. Ryckman et al. observed that mothers with preeclampsia had newborns with higher concentrations of acylcarnitines and free carnitine on their NBS compared to non-preeclamptic mothers.^11^ Jelliffe-Pawlowski et al. found that the NBS metabolites could predict gestational age and that size for gestational age was a variable that changed the predictive ability of their model.^10^ A range of studies have utilized NBS metabolites to predict newborn and pediatric conditions, with varying degrees of success.^2–11,34–36^ Our results extend the extant literature in several ways. NBS metabolites demonstrate a good ability to reflect infant metabolic alterations associated with diabetic and hypertensive disorders of pregnancy. As demonstrated in Fig. 1, the more severe the maternal phenotype condition, the greater the strength of association as predicted by the metabolite model and reflected by the AUROC for the specific condition. Type 1 diabetes and chronic hypertension with superimposed preeclampsia, considered among the more severe clinical phenotypes for their respective categories, had more robust predictions of metabolic disturbance (highest AUROC values). Although we did not have detailed information available on blood pressure ranges or glycemic control, our findings suggest that a greater degree of metabolic disruption in the neonate is present as the maternal clinical phenotype varies and becomes increasingly severe.

Since NBS analytes have been chosen by design to reflect metabolic disturbances associated with specific genetic and/or metabolic diseases, the analytes themselves robustly reflect alterations in specific lipid and protein metabolic pathways. Accordingly, through a detailed examination of the rank order of coefficients for those analytes associated with a specific maternal morbidity (Figs. 2 and 3), it is possible to gain greater insight to potentially altered biologic pathways in the newborn resulting from a given maternal morbidity and the implications thereof for clinical outcome risk. There are several leading features that are common to each of the maternal morbidity categories as reflected across the models. For diabetes, there were consistent associations with analytes C2, C3, C5, C5-OH, and free carnitine. Although not strongly observed in our investigation, other studies have demonstrated that branched chain (leucine, isoleucine, and valine) and aromatic amino acids (phenylalanine and tyrosine) are associated and in some instances predictive of future diabetes.^37^ For instance, it has been postulated that in obese individuals various metabolic signaling pathways lead to elevations in branched chain amino acids and mechanistic target of rapamycin signaling, which result in elevations in C3 and C5 acylcarnitines.^27,38^ C2 is also notable, as it associated with insulin resistance and directly correlates with hemoglobin A1C levels in adults, perhaps via its role in substrate switching at the level of the mitochondrial membrane.^29^ In sum, associations with short-chain acylcarnitines, C5-OH, and free carnitine in neonates born to diabetic mothers suggests that the metabolic signs of insulin resistance may already be present at birth. In fact, changes in nutrition status over long periods of time can create an imprint or “memory” of a metabolic disturbance that is passed down to offspring via germ-line epigenetic changes.^39–42^ Our investigation suggests that this effect may have been present for newborns exposed to maternal diabetes and is reflected in the NBS metabolites shortly after birth. Future studies that selectively examine infants from pregnancies with well and poorly controlled diabetes may help clinicians to understand, target, and design mitigating strategies to prevent short- and long-term sequelae of in utero hyperglycemia exposure.

Our results also suggest that there is a unique metabolic signature for infants born to mothers with preeclampsia with severe features that involves amino acids associated with the production and signaling of nitric oxide, a known vasodilator and mediator of blood pressure during pregnancy.^43^ While ornithine was positively predictive for infants born to mothers with preeclampsia with severe features and chronic hypertension with superimposed preeclampsia, citrulline was negatively predictive for infants born to any cohort of hypertensive disorders of pregnancy (Fig. 3). The role of nitric oxide formation and L-arginine metabolism in mothers with preeclampsia has received considerable attention as both a possible pathologic mechanism for preeclampsia and as a causal pathway for concomitant fetal growth restriction.^33,43^ Ornithine serves as a by-product of L-arginine via arginase, an important step in the urea cycle. L-arginine also serves as the substrate for nitric oxide synthase in the production of nitric oxide. Disturbances in the levels of vasodilators such as nitric oxide are known to cause endothelial dysfunction, which has been hypothesized as a possible leading cause of preeclampsia.^33,43^ There have been attempts to therapeutically modify this pathway via sildenafil administration albeit with limited success.^44,45^ In an analysis of all infants with SGA who were born to mothers with any form of preeclampsia (with or without severe features and/or superimposed on chronic hypertension, Fig. 5), citrulline was again negatively associated with SGA infants whose mothers had preeclampsia suggesting that there may be a biologic relationship between urea cycle intermediaries, preeclampsia, and SGA status.

The biologic mechanisms for growth altered fetuses affected by diabetes and hypertensive disorders have not been fully elucidated. There are, however, potentially significant clinical implications for gaining a deeper understanding of these mechanisms given that many affected fetuses and newborns later develop chronic health conditions as adolescents or adults.^24,46–52^ We observed several metabolites (Fig. 4) that were consistently associated with LGA newborns subject to maternal diabetes, including C5-OH, glycine, and long-chain acylcarnitines C14, C16, and C18. All of these metabolites have been previously linked with macrosomia, insulin resistance, or sensitivity.^26,27,29,53–55^ Long-chain acylcarnitines are of particular interest, as a preponderance of evidence suggests that they serve as upstream participants in pro-inflammatory pathways that help produce cyclooxygenase-2 and nuclear factor kB, resulting in macrophage activation and interleukin-6 release.^56–60^ Given that many acquired diseases of the neonate (NEC, BPD, patent ductus arteriosus, early-onset sepsis) are mediated via changes in these pro-inflammatory mediators, it is possible that elevations in long-chain acylcarnitines in LGA infants of diabetic mothers deleteriously impact an already sensitized inflammatory milieu. When examining SGA infants born to preeclamptic mothers (Fig. 5), we see a vastly different metabolite composition, which includes intermediates of nitric oxide formation (citrulline) and short-chain acylcarnitines, suggesting impaired lipid metabolism. Intriguingly, the metabolite associations for each maternal condition correspond with newborn size designations and are wholly unique to the designated condition and the associated newborn growth phenotype. Given that growth is an energy-dependent process, examining the metabolic function of the newborn may provide greater biologic insight into both the appropriately sized and growth altered fetus and thus allow for a more precise approach to risk stratify newborns as part of a clinical application of the fetal origins of childhood and adult disease hypothesis.

This is one of the largest studies to date using standardized NBS laboratory methods to assess the metabolic profile at birth of >40,000 newborns, with linked results to the presence or absence of maternal disease and corresponding newborn size designation at birth. However, there are a few limitations of this study. The sample population was not random as this was a population study that selected infants for specific diseases of prematurity. Because many mothers who had preeclampsia delivered prematurely, it is possible that the metabolites in these infants reflect metabolism at younger gestational ages. There are also some variables such as birth weight, exposure to total parenteral nutrition, blood transfusions, enteral intake, and timing of NBS collection that were not entirely controlled for. Further, maternal conditions such as diabetes and hypertensive disorders of pregnancy may contribute to both maternal and fetal oxidative or catabolic stress.^11,22,30^ Given that we could not measure such stress impacts, we cannot know whether our findings represent a direct or indirect functional relationship to a pathway involving oxidative and/or catabolic stress or a function of the specific maternal condition pathway directly. Additionally, given that analytes measured on the NBS are markers of dynamic metabolic processes that likely change over time, future studies will ideally assess the NBS metabolic profile at multiple time points during and after pregnancy to identify how maternal disease biometrics (e.g., blood pressure or glycemic control) change the analyte composition longitudinally. Moreover, our findings were derived from a case–control study, and thus there was an oversampling relative to the population base of preterm infants. To the extent that our prediction models are unique to preterm infants, we are not indicating specific metabolite screening cutoffs be inferred from this work. Future investigations on a population cohort are necessary to determine whether recommendations of metabolite cutoffs might be warranted. Lastly, given that the NBS is a clinical application of targeted metabolomics limited to a few dozen analytes designed to alert to the possibility of specific heritable diseases, we speculate that future non-targeted metabolomic studies capable of examining hundreds to thousands of additional analytes will reveal ever greater biologic insight and clinical precision for applications in maternal and neonatal disease.

## Supplementary Material

Supplementary Material

## IMPACT:

- Substantial differences in newborn screen analyte profiles were present based on the presence or absence of maternal diabetes or hypertensive disorder of pregnancy and this finding was further influenced by the newborn size designation at birth.
- The metabolic health of the newborn can be examined using the newborn screen and is heavily impacted by the condition of the mother during pregnancy.
- Utilizing the newborn screen to identify newborns affected by common conditions of pregnancy may help relate an infant’s underlying biological disposition with their clinical phenotype allowing for greater risk stratification and intervention.
