# Supplementary material for: Newborn screen metabolic panels reflect the impact of common disorders of pregnancy
Source: Pediatr Res. Author manuscript; Available in PMC 2023 Aug 1. (PMC10265936; doi:10.1038/s41390-021-01753-7)
Supplement: Supplementary Material [file NIHMS1902998-supplement-Supplementary_Material.pdf]

<u>Supplemental Material</u>

Supplemental Table 1. Newborn Screen Analyte Abbreviations

Supplemental Table 2. Maternal Conditions Definitions

Supplemental Table 3. Demographic Information for Mother-Infant Dyads with and without Maternal Diabetes during Pregnancy

Supplemental Table 4. Demographic Information for Mother-Infant Dyads with and without Hypertensive Disorders of Pregnancy

Supplemental Figure 1. Inclusion Criteria

Supplemental Table 1. Newborn Screen Analyte Abbreviations

**Newborn Screen Analyte**	**Abbreviation**
Free Carnitine	FC
Acetylcarnitine	C2
Propionylcarnitine	C3
Malonylcarnitine	C3-DC
Butyrylcarnitine+Isobutyrylcarnitine	C4
Isovalerylcarnitine + Methylbutyrlcarnitine	C5
Tiglylcarnitine	C5:1
Glutarylcarnitine	C5-DC
Hexanoylcarnitine	C6
Octanoylcarnitine	C8
Octenoylcarnitine	C8:1
Decanoylcarnitine	C-10
Decenoylcarnitine	C10:1
Dodecanoylcarnitine	C12
Dodecenoylcarnitine	C12:1
Tetradecanoylcarnitine	C14
Tetradecenoylcarnitine	C14:1
3-Hydroxytetradecadienoylcarnitine	C14OH
Palmitoylcarnitine	C16
3-Hydroxypalmitoylcarnitine	C16OH
Palmitoleylcarnitine	C16:1
Stearoylcarnitine	C18
Oleoylcarnitine	C18:1
Linoleoylcarnitine	C18:2
3-Hydroxyoleoylcarnitine	C18:1OH
**Amino Acids**	
Alanine	ALA
Arginine	ARG
Citrulline	CIT
Glycine	GLY
Methionine	MET
Ornithine	ORN
Phenylalanine	PHE
Proline	PRO

5-Oxoproline	OXP
Succinylacetone	SA
Tyrosine	TYR
Valine	VAL
Leucine	LEU
Isoleucine	ISO

Supplemental Table 2. Maternal Conditions Definitions

**Maternal Condition**	**Definition**
Gestational diabetes	ICD9: 648.8
Type I diabetes	ICD9: 250.x1, 250.x3
Type II diabetes	ICD9: 250.x0, 250.x2, 648.0
Chronic hypertension	ICD9: 401-405, 642.0-642.2, 642.9
Gestational hypertension	ICD9: 642.3
Preeclampsia without severe features	ICD9: 642.4
Preeclampsia with severe features	ICD9: 642.5, 642.6
Preeclampsia (any form) superimposed on chronic hypertension	ICD9: 642.7, or any chronic hypertension code combined with any preeclampsia code

Abbreviation: ICD9, International Statistical Classification of Diseases and Related Health Problems, 9th edition

Supplemental Table 3. Demographic Information for Mother-Infant Dyads with and without Maternal Diabetes during Pregnancy

	Type 1 Diabetes	Type 2 Diabetes	Gestational Diabetes	No Diabetes
	n = 126 (%)	n = 481 (%)	n = 2752 (%)	n = 37974 (%)
**Maternal Race/Ethnicity**				
Non-Hispanic White	45 (35.7)	64 (13.3)	485 (17.6)	9,956 (26.2)
Non-Hispanic Black	16 (12.7)	44(9.2)	165 (6.0)	3,124 (8.2)
Asian	8 (6.4)	55 (11.4)	548 (19.9)	4,124 (10.9)
Pacific Islander	2 (1.6)	11 (2.3)	19 (0.7)	196 (0.5)
Hispanic	52 (41.3)	303 (63.0)	1,512 (54.9)	20,299 (53.5)
Native American/Native Alaskan	1 (0.8)	1 (0.21)	4 (0.2)	63 (0.2)
Other	1 (0.8)	---	---	13 (0.03)
Missing	1 (0.8)	3 (0.6)	19 (0.7)	199 (0.5)
**Delivery Method**				
Vaginal	47 (37.3)	125 (26.0)	1,317 (47.9)	22,424 (59.1)
Cesarean section	79 (62.7)	356 (74.0)	1,435 (52.1)	15,550 (40.9)
**Gestational Age, weeks**				
22-23	3 (2.4)	7 (1.5)	21 (0.8)	647 (1.7)
24-25	19 (15.1)	49 (10.2)	128 (4.7)	2708 (7.1)
26-27	24 (19.1)	101 (21)	283 (10.3)	3754 (9.9)
28-29	40 (31.8)	124 (25.8)	530 (19.3)	5072 (13.4)
30-31	0 (0)	7 (1.5)	9 (0.3)	63 (0.2)
32-33	2 (1.6)	6 (1.3)	20 (0.7)	211 (0.6)
34-35	5 (3.9)	10 (2.1)	64 (2.3)	631 (1.7)
36	4 (3.2)	14 (2.9)	86 (3.1)	811 (2.1)
37-38	15 (11.9)	71 (14.8)	632 (22.9)	7154 (18.8)
39-40	13 (10.3)	87 (18.1)	881 (32.)	14290 (37.6)
41-42	1 (0.8)	4 (0.8)	91 (3.3)	2452 (6.5)
43-44	0 (0)	1 (0.2)	7 (0.2)	181 (0.5)
**Birthweight (g)**				
0-499	2 (1.6)	6 (1.2)	11 (0.4)	303 (0.8)
500 - 999	48 (38.1)	139 (28.9)	362 (13.1)	6326 (16.7)
1000 - 1499	24 (19.0)	108 (22.4)	465 (16.9)	4681 (12.3)
1500 - 1999	12 (9.5)	29 (6.0)	127 (4.6)	896 (2.4)

2000 - 2499	5 (3.9)	18 (3.7)	94 (3.4)	1017 (2.7)
2500 - 2999	12 (9.5)	28 (5.8)	317 (11.5)	4414 (11.6)
3000 - 3499	8 (6.3)	72 (14.9)	679 (24.7)	10670 (28.1)
3500 - 3999	11 (8.7)	51 (10.6)	503 (18.3)	7553 (19.9)
4000 - 4499	3 (2.4)	23 (4.8)	150 (5.4)	1843 (4.8)
4500 - 4999	1 (0.8)	5 (1.0)	39 (1.4)	250 (0.6)
5000 - 5499	0 (0)	1 (0.2)	5 (0.2)	17 (0.04)
5500 - 5999	0 (0)	0 (0)	0 (0)	4 (0.01)
6000 - 6049	0 (0)	1 (0.2)	0 (0)	0 (0)
**Infant Sex**				
Male	66 (52.4)	270 (56.1)	1,489 (54.1)	19,666 (51.8)
Female	60 (47.6)	211 (43.9)	1,263 (45.9)	18,308 (48.2)
**Mortality**				
Survived	114 (90.5)	447 (92.9)	2,674 (97.2)	36,445 (95.9)
Death < 28 days	12 (9.5)	16 (3.3)	52 (1.9)	1,017 (2.7)
Death > 28 days	---	18 (3.7)	26 (0.9)	512 (1.4)
**Size for Gestational Age Designation[a]**	**Type 1 Diabetes**	**Type 2 Diabetes**	**Gestational Diabetes**	**No Diabetes**
	**n = 126 (%)**	**n = 481 (%)**	**n = 2752 (%)**	**n = 37974 (%)**
Small for Gestational Age	31 (24.8)	118 (24.8)	429 (16.2)	5,837 (16.5)
Appropriate for Gestational Age	64 (51.2)	239 (50.2)	1,642 (61.9)	24,103 (68.2)
Large for Gestational Age	30 (24.0)	119 (25.0)	583 (21.9)	5,401 (15.3)

[a] Size for Age Designations based on World Health Organization definitions of normative data for fetal size. Designations not available for infants 41 - 44 weeks.

Supplemental Table 4. Demographic Information for Mother-Infant Dyads with and without Hypertensive Disorders of Pregnancy

	Chronic HTN	Gestational HTN	PE without SF	PE with SF	Chronic HTN with Superimposed PE	No HTN Disorder of Pregnancy
	n = 777 (%)	n = 756 (%)	n = 767 (%)	n = 1848 (%)	n = 845 (%)	n = 36340 (%)
**Maternal Race/Ethnicity**						
Non-Hispanic White	204 (26.3)	208 (27.5)	182 (23.7)	436 (23.6)	151 (17.9)	9,369 (25.8)
Non-Hispanic Black	132 (17.0)	92 (12.2)	69 (9.0)	228 (12.3)	143 (16.9)	2,685 (7.4)
Asian	88 (11.3)	64 (8.5)	64 (8.3)	165 (8.9)	105 (12.4)	4,249 (11.7)
Pacific Islander	10 (1.30)	3 (0.4)	6 (0.8)	13 (0.7)	11 (1.3)	185 (0.5)
Hispanic	334 (43.00)	386 (51.1)	444 (57.9)	970 (52.5)	411 (48.6)	19,621 (53.99)
Native American/Native Alaskan	4 (0.5)	1 (0.1)	---	9 (0.5)	6 (0.7)	49 (0.1)
Other	---	---	---	---	---	14 (0.04)
Missing	5 (0.6)	2 (0.3)	2 (0.3)	27 (1.5)	18 (2.1)	168 (0.5)
**Delivery Method**						
Vaginal	297 (38.2)	362 (47.9)	343 (44.7)	152 (8.2)	47 (5.6)	22,712 (62.5)
Cesarean section	480 (61.8)	394 (52.1)	424 (55.3)	1,696 (91.8)	798 (94.4)	13,628 (37.5)
**Gestational Age, weeks**						
22-23	13 (1.67)	1 (0.1)	0 (0)	20 (1.1)	7 (0.8)	637 (1.7)
24-25	88 (11.3)	23 (3.0)	18 (2.3)	188 (10.2)	105 (12.4)	2482 (6.8)
26-27	150 (19.3)	39 (5.2)	59 (7.7)	491 (26.6)	256 (30.3)	3167 (8.7)
28-29	165 (21.2)	113 (14.9)	140 (18.2)	893 (48.3)	396 (46.9)	4059 (11.2)
30-31	5 (0.6)	1 (0.1)	1 (0.1)	14 (0.8)	1 (0.1)	57 (0.2)
32-33	6 (0.7)	10 (1.3)	7 (0.9)	26 (1.4)	6 (0.7)	184 (0.5)
34-35	18 (2.3)	23 (3.0)	34 (4.4)	48 (2.6)	15 (1.8)	572 (1.6)
36	20 (2.6)	27 (3.6)	44 (5.7)	30 (1.6)	7 (0.8)	787 (2.2)
37-38	123 (15.8)	209 (27.6)	194 (25.3)	73 (3.9)	33 (3.9)	7240 (19.9)
39-40	174 (22.4)	257 (34.0)	229 (29.9)	56 (3.0)	18 (2.1)	14537 (40)
41-42	13 (1.6)	50 (6.6)	37 (4.8)	9 (0.5)	1 (0.1)	2438 (6.7)

43-44	2 (0.3)	3 (0.4)	4 (0.5)	0 (0)	0 (0)	180 (0.5)
**Birthweight (g)**						
0-499	18 (2.3)	8 (1.1)	7 (0.9)	72 (3.9)	51 (6.0)	166 (0.5)
500 - 999	242 (31.1)	85 (11.2)	113 (14.7)	955 (51.7)	457 (54.1)	5023 (13.8)
1000 - 1499	140 (18.0)	61 (8.1)	81 (10.6)	523 (28.3)	230 (27.2)	4243 (11.7)
1500 - 1999	25 (3.2)	30 (3.9)	28 (3.6)	92 (4.9)	34 (4.0)	855 (2.3)
2000 - 2499	33 (4.2)	43 (5.7)	56 (7.3)	63 (3.4)	13 (1.5)	926 (2.5)
2500 - 2999	80 (10.3)	122 (16.1)	145 (18.9)	59 (3.2)	20 (2.4)	4345 (11.9)
3000 - 3499	126 (16.2)	209 (27.6)	185 (24.1)	60 (3.2)	24 (2.8)	10825 (29.8)
3500 - 3999	84 (10.8)	148 (19.6)	113 (14.7)	18 (0.9)	15 (1.7)	7740 (21.3)
4000 - 4499	26 (3.3)	38 (5.0)	35 (4.6)	6 (0.3)	0 (0)	1914 (5.3)
4500 - 4999	3 (0.4)	9 (1.2)	3 (0.4)	0 (0)	1 (0.1)	279 (0.8)
5000 - 5499	0 (0)	2 (0.2)	1 (0.1)	0 (0)	0 (0)	20 (0.1)
5500 - 5999	0 (0)	1 (0.1)	0 (0)	0 (0)	0 (0)	3 (0.01)
6000 - 6049	0 (0)	0 (0)	0 (0)	0 (0)	0 (0)	1 (0.00)
**Infant Sex**						
Male	401 (51.6)	408 (53.9)	399 (52.0)	897 (48.5)	396 (46.9)	18,990 (52.3)
Female	376 (48.4)	348 (46.0)	368 (48.0)	951 (51.5)	449 (53.1)	17,350 (47.7)
**Mortality**						
Survived	715 (92.0)	735 (97.2)	750 (97.8)	1,712 (92.6)	783 (92.7)	34,985 (96.3)
Death < 28 days	35 (4.5)	15 (1.9)	9 (1.2)	78 (4.2)	33 (3.9)	927 (2.6)
Death > 28 days	27 (3.5)	6 (0.8)	8 (1.0)	58 (3.1)	29 (3.4)	428 (1.2)
**Size for Gestational Age Designation**[a]						
Small for Gestational Age	228 (29.9)	188 (26.7)	221 (30.4)	1,151 (62.6)	529 (62.7)	4,098 (12.2)
Appropriate for Gestational Age	428 (56.2)	397 (56.5)	406 (55.9)	572 (31.1)	267 (31.6)	23,978 (71.1)
Large for Gestational Age	106 (13.9)	118 (16.8)	99 (13.6)	116 (6.3)	48 (5.7)	5,646 (16.7)

[a] Size for Age Designations based on World Health Organization definitions of normative data for fetal size. Designations not available for infants 41 - 44 weeks.

Supplemental Figure 1. Inclusion Criteria

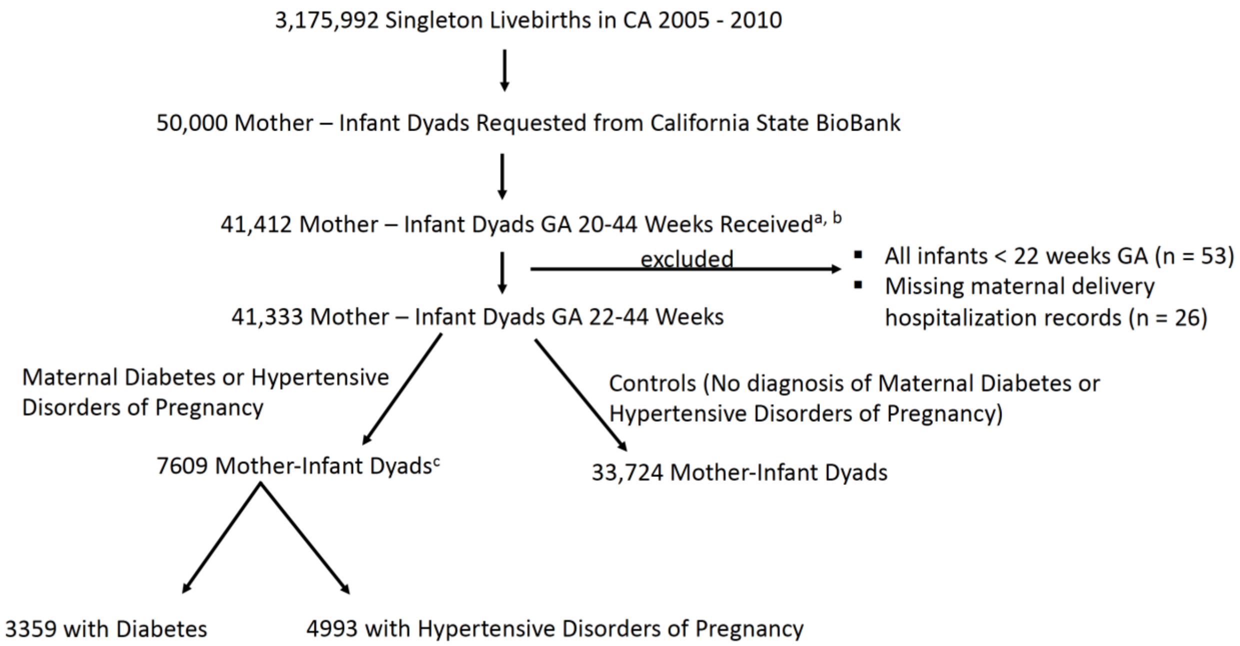

[a] NBS metabolite data from California State BioBank merged with OSHPD and CPQCC records

[b] Dyads selected from population study of 20-29 week infants with neonatal outcomes (IVH, NEC, BPD, ROP) and from random sample of births from GA 30-44 weeks.

[c] 743 mothers with both Diabetes and concurrent Hypertensive Disorder of Pregnancy
